# Comparative Assessment of the Nutritional Profile of Meat Products and Their Plant-Based Analogues

**DOI:** 10.3390/nu15122807

**Published:** 2023-06-19

**Authors:** Judit Costa-Catala, Natalia Toro-Funes, Oriol Comas-Basté, Salvador Hernández-Macias, Sònia Sánchez-Pérez, M. Luz Latorre-Moratalla, M. Teresa Veciana-Nogués, Victòria Castell-Garralda, M. Carmen Vidal-Carou

**Affiliations:** 1 Departament de Nutrició, Ciències de l’Alimentació i Gastronomia, Facultat de Farmàcia i Ciències de l’Alimentació, Campus de l’Alimentació de Torribera, Universitat de Barcelona, Av. Prat de la Riba 171, 08921 Santa Coloma de Gramenet, Spain; jcostacatala@ub.edu (J.C.-C.); soniasanchezperez@ub.edu (S.S.-P.); mariluzlatorre@ub.edu (M.L.L.-M.); mcvidal@ub.edu (M.C.V.-C.); 2 Institut de Recerca en Nutrició i Seguretat Alimentària (INSA·UB), Universitat de Barcelona (UB), Av. Prat de la Riba 171, 08921 Santa Coloma de Gramenet, Spain; 3 Facultad de Ciencias de la Salud, Universidad Internacional de Valencia (VIU), C/Pintor Sorolla 21, 46002 Valencia, Spain; natalia.toro@campusviu.es; 4 Departamento de Salud Pública, Centro Universitario de Ciencias Biológicas y Agropecuarias, Universidad de Guadalajara, Camino Ramón Padilla Sánchez 2100, Zapopan 45200, Mexico; salvador.hernandez@cucba.udg.mx; 5 Servei de Planificació, Auditoria i Avaluació del Risc i Comunicació, Departament de Salut, Generalitat de Catalunya, C/Roc Boronat 81-95, 08005 Barcelona, Spain; victoria.castell@gencat.cat

**Keywords:** plant-based meat analogues, nutrient profile, ingredients, nutrition labeling, plant-based protein, food labeling

## Abstract

Vegetarian and vegan diets are increasingly being adopted in Spain, a trend mainly driven by ethical concerns for animal welfare and the environment. This has resulted in a growing market for plant-based substitutes of meat products. However, available data on the nutritional value of such meat analogues in Mediterranean countries are still limited. In this study, the labelling information of four categories of plant-based meat analogues (*n* = 100) and the corresponding conventional meat products (*n* = 48) available on the Spanish market was surveyed and compared. The nutrient content of plant-based meat analogues varied significantly, due to the wide range of ingredients used in their formulation. Some of these products were found to have a low protein content, which in others was enhanced by the addition of cereals and legumes. Compared to the meat products, the plant-based analogues contained lower levels of total fat as well as saturated fat, which ranged from 30% of total fat in burgers to less than 15% in meatballs, sausages, and nuggets; in contrast, they contained higher amounts of fiber and complex carbohydrates. Overall, the meat analogues cannot be considered as nutritionally equivalent substitutes to conventional meat products due to a high variability of protein content and other nutrients.

## 1. Introduction

Vegetarianism and veganism are growing trends in Spain: according to data published by the Lantern consultancy in 2021, the number of people following a vegetarian or vegan diet increased by 34% in 2019–2021, compared to a growth of 27% in the previous biennium [1,2]. Principle causes driving this tendency are ethical concerns for animal welfare and the environment, as well as the belief that these diets are healthier than those including products of animal origin. In parallel with these trends, the availability of meat analogues on the market has also increased.

Tofu, a soy-based product used as a meat substitute, was first consumed in China. However, the first patent for soy protein textured to resemble meat was issued in the United States in 1955 [3]. Until 1960, the consumption of tofu and soy protein in Western countries was very limited, but since then it has experienced continual growth. Between 2015 and 2021, 4400 products made from plant ingredients were launched on the market as substitutes for different products of animal origin. The global market for meat analogues is currently dominated by European countries, principally the United Kingdom, Germany, Italy, France, Sweden, Belgium, Norway, and the Netherlands, which are at the forefront of innovation in the sector of meat protein alternatives [4].

In a study carried out by ProVeg International, it is estimated that in Spain the sales volume of plant-based products analogous to foods of animal origin has increased by 20% in the last two years, with plant-based “milks”, “meat”, and “yogurt” leading the market [5]. According to this report, consumers in Spain are open to buying plant-based meat substitutes more regularly than in other countries. Thus, for example, 47% of Spanish consumers would be willing to purchase these products if they had the same flavor and texture as the equivalent meat and dairy foods [5].

From a health point of view, reducing consumption of products of animal origin, especially red meat, and substituting them for foods of plant origin has been associated with lower overall mortality in epidemiological studies [6]. However, the health benefits of following a plant-based diet cannot be directly extrapolated to the replacement of meat products with plant-based analogues. Little is known about the composition of these substitutes, whose formulations are highly variable. They are frequently produced from vegetable protein concentrates and isolates and the presence of other plant components (e.g., vitamin C, carotenoids, polyphenols) is not guaranteed.

Studies have addressed the nutritional composition of plant-based meat analogues marketed in different countries, such as Australia [7], the United States [8,9,10], and Colombia [11]. However, despite the unprecedented growth that meat substitutes have recently experienced in the European market, little has been published on their nutritional composition in comparison with conventional meat products. Some studies have compared the nutritional profile of different plant-based meat alternatives marketed in Europe [12,13,14,15,16], but nutritional information about such products sold in Mediterranean countries, including Spain, is limited. The first composition table of plant-based meat analogues marketed in Spain was published recently [17], but a comparative analysis of substitute and conventional products in terms of nutrition has not been previously carried out. To address this gap, the aim of the present study was to perform a comparative assessment of the nutritional profile of plant-based meat analogues available in Spain in comparison with the equivalent meat products.

## 2. Materials and Methods

### 2.1. Data Collection

A total of 148 products retailed in supermarkets and small shops in Barcelona (Spain) were evaluated: 100 plant-based meat analogues and 48 meat equivalents, grouped into four categories. These were burgers (25 plant-based and 25 animal-based), meatballs (25 plant-based and 9 animal-based), sausages (25 plant-based and 8 animal-based), and nuggets (25 plant-based and 7 animal-based).

We reviewed the information on product labels about ingredients, nutritional composition (calories, protein, total fat, saturated fat, carbohydrates, fiber, sugars, and salt), and allergens, as well as any claim of nutritional and health properties and marks of quality referring to suitability for vegetarians and vegans or the production process (organic/non-organic).

### 2.2. Statistical Analysis

The nutrient composition per 100 g of plant-based and animal-based products of the same category was compared using the Mann–Whitney *U* test. The analysis of variance among products of the same category was performed using the Kruskal–Wallis test. Statistical analysis was carried out with IBM SPSS Statistics 27.0 statistical software package (IBM Corporation, Armonk, NY, USA). Values of *p* < 0.05 were considered significant.

## 3. Results and Discussion

Table 1 shows the mean, maximum, and minimum number of ingredients listed on the labels of the four categories of plant-based and animal-based products. Both types of products were found to contain a high number of ingredients. The plant-based burgers and sausages contained a very similar mean number of ingredients, which was higher in plant-based meatballs and nuggets, which appear to have a more complex formulation. In addition, a high variability in the number of ingredients was also observed. For example, it can be found plant-based burgers formulated from nine ingredients to others made with 22 ingredients. According to the literature, plant-based meat analogues generally contain a long list of ingredients [3], a tendency that was confirmed in the present study, although they did not show statistically significant differences from the meat equivalents in this respect (*p* > 0.05). In fact, in the case of meat nuggets, the mean number of ingredients was 17 compared to 16 in the plant-based analogues. Moreover, the highly variable number of ingredients in products of the same category, whether of plant or animal origin, precludes any generalization in this respect. The term “ultra-processed” has recently been proposed to describe food products typically with five or more and usually many ingredients, mostly not commonly used in culinary preparations or classes of additives designed to make the final product palatable or more attractive [18,19]. Although this term has not been legally defined in Europe, the data we have collected suggest that both the animal- and plant-based foods surveyed could be included under this concept.

Supplementary Table S1 gives the ingredients listed on the labels of plant-based burgers, meatballs, sausages, and nuggets in descending order of frequency. The most common ingredient overall is salt, which is listed in more than 90% of each one the products analyzed. In second place are water and spices, which are found in 20 of the 25 products in each of the four categories, whereas the other ingredients have a variable presence.

**Figure 1:**
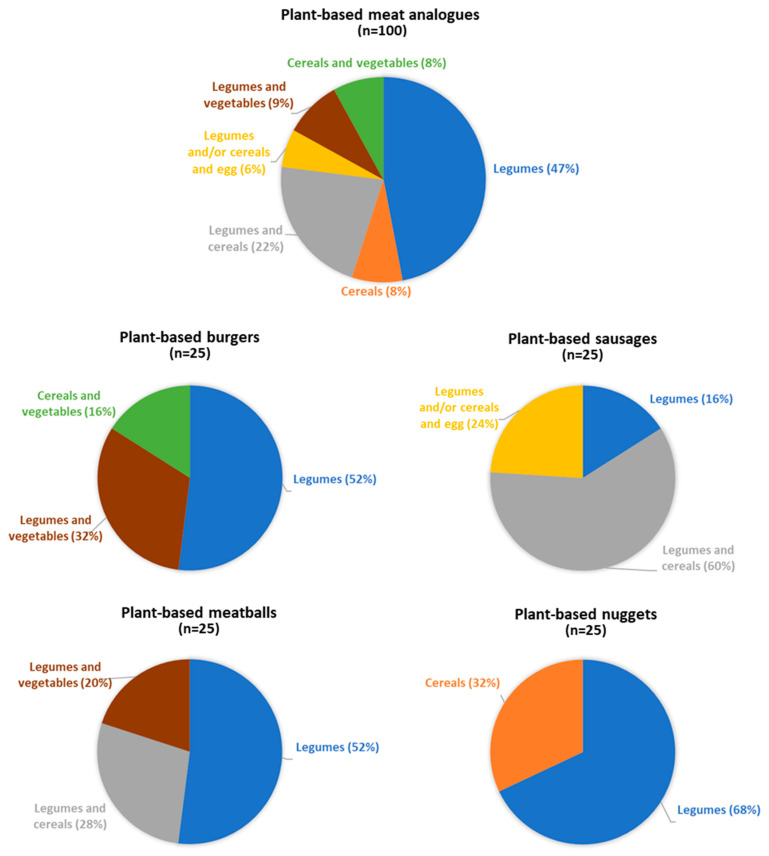
Percentage of plant-based meat analogues according to the main ingredients used as protein source. Data is shown for all meat analogues and for each individual category (burgers, meatballs, sausages, and nuggets).

Figure 1 shows the main ingredients used as plant-based protein source of the meat analogues, as well as for each individual category. Almost half of all products were just made with legumes as the main ingredient, with soy being the most frequent (used in the form of soy, tofu, texturized soybean protein, or soybean flour). In addition to soybeans, some meat analogues used other legumes, such as pea protein (18%) and chickpea protein (9%). The use of these two legumes to formulate meat analogue products, together with lentils and lupines, has notably increased in recent times [3]. An interesting feature of pea protein is that it is less allergenic than soy protein. Only 8% of all plant-based products were made with cereals as the main ingredient, mainly oats, wheat, and rice. Only 22% of meat analogues contained a mixture of legumes and cereals. It is well-established that proteins of cereals and legumes complement each other, as the deficiency of sulfur amino acids of legumes is covered by cereals and the deficiency of lysine in cereals is compensated by legumes. Moreover, 17% of the plant-based meat analogues included vegetables in their formulation based on legumes and/or cereals (e.g., onion, carrot, tomato, spinach, mushroom, eggplant, beetroot, and pumpkin). Some vegetables can contribute to maintaining or improving the flavor and the color of the final product. Soy or gluten proteins show a natural color ranging from pale yellow to beige or brown. This natural color is far from the reddish color that the consumer associates with a raw meat product. The color of food can be modified with some vegetables, like beetroot extracts, which contain betaine; carrots, with beta-carotene; tomato, with lycopene; or berries or raisins, rich in anthocyanins. When stratifying according to the different categories of meat analogues, while legumes continued to be the main ingredient in most products (with the exception of plant-based sausages, in which legumes are mostly combined with cereals), a non-homogeneous formulation profile was observed among plant-based burgers, sausages, meatballs, and nuggets. The ingredient composition of each plant-based meat analogue will be individually considered in the subsequent sections.

### 3.1. Comparative Analysis of the Nutritional Profile of Plant-Based Meat Analogues and Their Meat Equivalents

**Figure 2:**
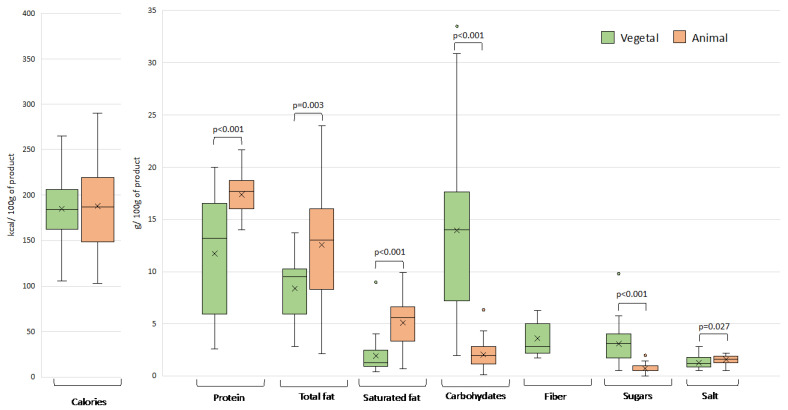
Energy value and nutrient content in plant- and animal-based burgers. Outliers are plotted as circles and the “x” represents the mean. Significant differences between the two types of products for the different nutritional parameters are shown.

**Figure 3:**
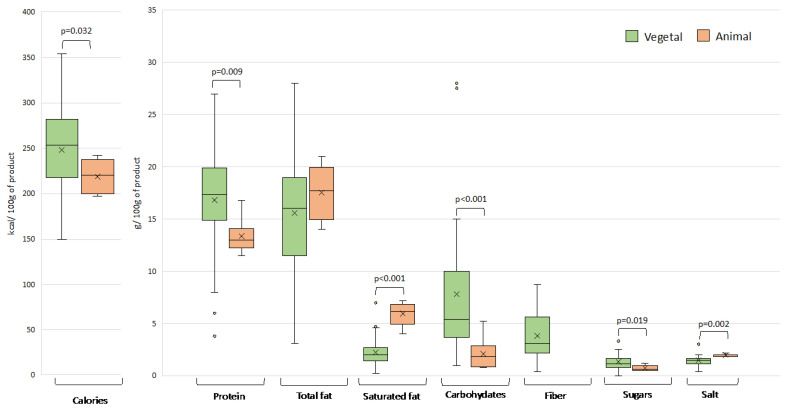
Energy value and nutrient content in plant- and animal-based sausages. Outliers are plotted as circles and the “x” represents the mean. Significant differences between the two types of products for the different nutritional parameters are shown.

**Figure 4:**
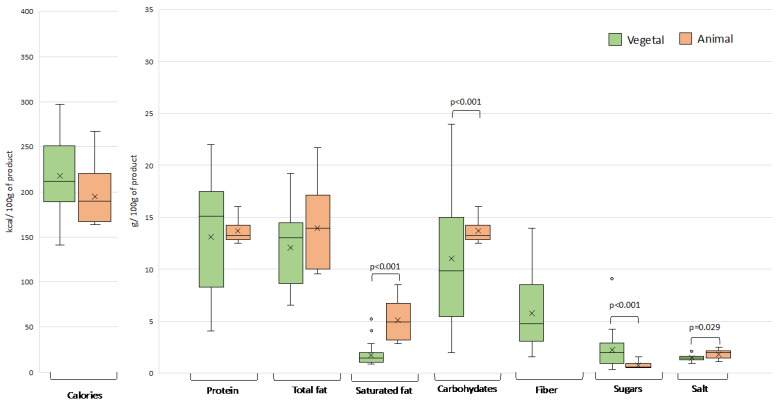
Distribution of energy and nutrients in plant- and animal-based meatballs. Outliers are plotted as circles and the “x” represents the mean. Significant differences between the two product types for the different nutritional parameters are shown.

**Figure 5:**
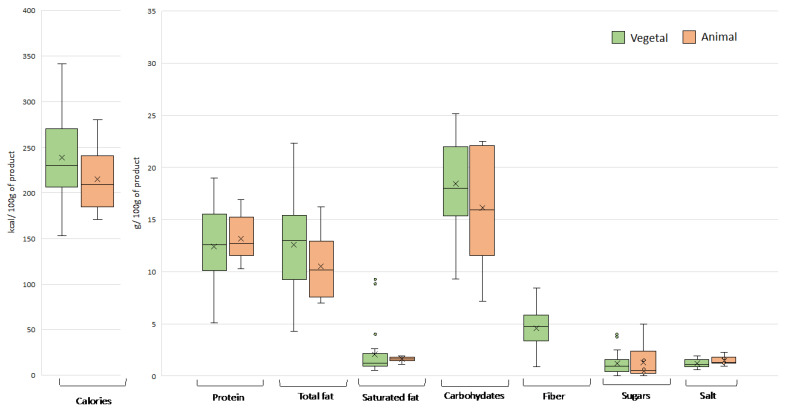
Energy value and nutrient content in plant- and animal-based nuggets. Outliers are plotted as circles and the “x” represents the mean.

The energy and nutrient content of plant-based meat analogues on the Spanish market was found to vary considerably, even among products of the same category, with a coefficient of variation that in several cases approached or exceeded 100% (Figure 2, Figure 3, Figure 4 and Figure 5). This variability can be explained by the wide range of ingredients and formulations used in their preparation. Similar differences were also observed in the products of animal origin, partly due to the different proportion of meat used in their formulation (ranging from 33% to 100%). A high diversity in nutritional composition has also been reported for meat analogues marketed in other European countries, Australia, and the USA [7,8,14,15,16].

#### 3.1.1. Burgers

Figure 2 shows the distribution of energy and nutrients in plant- and animal-based burgers. Contrary to the perception of many consumers about a lower caloric content of plant-based meat analogues, the two types have very similar caloric values (185.2 kcal/100 g and 187.9 kcal/100 g for plant- and animal-based products, respectively). In a study on burgers marketed in the European Union, Boukid and Castellari [12] reported that the energy values of plant-based burgers were similar to those of fish and pork burgers but lower compared to beef burgers.

The plant-based burgers were found to have a statistically lower mean protein content than those of animal origin (*p* < 0.001). Thus, if a vegetable burger is consumed as an alternative source of protein to replace a high-protein food (a meat burger), the daily intake of protein could be compromised, requiring compensation in other meals or consumption of a larger burger. It should be noted that the protein content in plant-based burgers is more variable than in the meat counterparts due to the variety of the main ingredients used for their preparation.

The main ingredients of the plant-based burgers evaluated in this study were legumes, cereals plus vegetables, or legumes plus vegetables (Figure 1). Legumes (15.0 g/100 g) afforded a significantly higher protein content than legumes plus vegetables (10.1 g/100 g) or cereals plus vegetables (5.4 g/100 g). Notably, soy protein concentrates can reach a protein richness of up to 70% [20]. These concentrates, often incorporated as textured or non-textured protein blends, provide the stringy texture and mouthfeel of authentic meat [21,22]. Developing plant-based products with sensory properties (taste, aroma, and texture) similar to meat to achieve consumer acceptance is challenging. Several studies have shown that the sensory acceptability of plant-based meat analogues by consumers is generally lower than meat or meat products [23,24]. According to the analysis of consumer preferences performed by Profeta et al. in Germany and Belgium, more than 60% of respondents considered meat tastier compared to plant-based meat analogues, mainly focusing on texture [25]. In the same line, experiments under blind conditions seem to demonstrate that meat products are strongly preferred over plant-based analogues in terms of sensory characteristics [26]. According to this same study, when compared to meat burgers, plant-based burgers had an unpleasant taste and were perceived as less juicy, dry, and grainy. In fact, texture attributes, such as fibrous structure, tenderness, and juiciness of meat protein, are very difficult to recreate in plant-based analogues [24]. The use of soy proteins, as well as pea proteins, can enhance the acceptability of the textural properties of meat analogues [23,24]. Similarly, the application of 3D printing technologies, which can produce soy protein-based meat analogues with customized shapes, colors, flavors, and textures, may offer solutions [27]. Thus, soy is a valuable ingredient not only for its nutritional content, with soy seeds and flours being rich in protein, unsaturated fat, soluble and insoluble fiber, and micronutrients such as B vitamins or vitamin E, but also for technological reasons. Actually, soy protein has long been incorporated as an ingredient in conventional processed meat products, as it enhances water retention and juiciness after cooking.

The average value of total fat and saturated fat was higher in burgers of animal origin (12.6 g total fat and 5.1 g saturated fat/100 g) than in plant-based burgers (8.4 g total fat and 1.9 g of saturated fat/100 g). In general, vegetable foods are less fatty, with the exception of oils, nuts, oilseeds, and some oily fruits, such as olives. Saturated fat levels in the plant-based burgers were lower in absolute terms and represented a lower percentage of the total fat (on average 30%) compared to burgers of animal origin (40%). The fat content in the burger analogues seemed to be unaffected by the main ingredients used in their preparation.

The carbohydrate content of plant-based burgers varied considerably, but was generally higher compared to conventional burgers. In addition, the plant-based burgers contained dietary fiber (3.5 g/100 g). The sugar content was higher in plant-based than animal-based burgers (3.1 g/100 g versus 0.7 kcal/100 g, respectively). Within plant-based burgers, the sugar content was significantly higher (*p* < 0.05) in products containing cereals plus vegetables as the main ingredients.

Finally, the salt content was significantly lower in burgers of plant rather than animal origin (*p* < 0.027), which could be expected, given the lower microbiological stability of meat raw materials and the preservative properties of salt. In those of vegetable origin, salt is used mainly for organoleptic reasons. Nevertheless, none of the burger analogues could be labeled as low-salt products. Boukid and Cartellari [12] did not find statistically significant differences in the salt content between plant- and animal-based burgers, and other authors have even found higher salt content in meat-like vegetable products than in their meat counterparts [7,11].

#### 3.1.2. Sausages

Plant-based sausages were far more variable in terms of energy value (kcal) and nutrient content than the meat equivalents (Figure 3). This variability would be due to the differences in their formulation, with some containing legumes as the main ingredient, others containing legumes plus cereals, and a significant percentage (24%) also including eggs, which would not be suitable for vegans. Sausages were the only food category in which the energy value of plant (247.9 kcal/100 g) and animal (219.0 kcal/100 g) products differed with statistical significance (*p* = 0.032).

In contrast with burgers, some plant-based sausages had a significantly higher protein content than the meat equivalents (*p* < 0.009), whereas others showed no difference. A significant proportion of the plant-based sausages contained both soy and wheat gluten. Wheat, or, more specifically, wheat gluten, is another source of protein in plant-based meat analogues [3]. The viscoelastic properties of wheat gluten and the relative simplicity of its extraction from flour make it an ideal ingredient for the formulation of products seeking to mimic meat [28].

Sausages formulated from legumes or legumes plus cereals had a significantly higher average protein content (17.2 g/100 g and 18.8 g/100 g, respectively) than those also formulated with eggs (7.0 g/100 g). Although eggs can be considered a protein food par excellence, the amount found in some sausages suggests they are added as an emulsifier and not to considerably increase the protein content.

Although there were no differences in the total fat content between plant-based sausages and those of animal origin, the level of saturated fat in the former was statistically lower (*p* < 0.001). In fact, the percentage of total fat that was saturated in plant-based sausages was even lower than in plant-based burgers (less than 15% on average). These differences are clearly attributable to the content of vegetable fats (68% of the plant-based sausages contained sunflower oil and the rest rapeseed oil or shea butter), which are richer in mono- and polyunsaturated fatty acids than animal fats.

The carbohydrate and sugar values were significantly higher in the plant-based sausages due to the content of legumes and cereals (*p* < 0.001 and *p* = 0.019, respectively). Although these ingredients can be considered as essentially a source of protein, they also contribute to the final carbohydrate content, as do breadcrumbs and various other ingredients, although to a lesser extent. Some of the sausage analogues were found to contain starches used as thickeners or substitutes for hydrocarbon-based lipids.

Finally, plant- and animal-based sausages differed in salt content (*p* = 0.002). It should be noted that the salt content was clearly higher in sausage analogues formulated with eggs than without (3.0 g/100 g and 1.4 g/100 g, respectively).

#### 3.1.3. Meatballs

Like burgers, plant-based and conventional meatballs did not differ significantly in terms of energy value (Figure 4). However, unlike in burgers and sausages, no significant differences in protein content were observed between the two types of meatballs. The main ingredient of the meatball analogues was legumes, either alone or together with vegetables or cereals (Figure 1). The average protein content of those containing legumes plus vegetables was almost 6 g/100 g, being statistically significantly lower compared to the other two formulations, which reached average values of around 12–15 g/100 g (*p* < 0.05). In conventional meatballs, the average protein content was close to 14 g/100 g, so the only plant-based meatballs with a clearly lower protein content were those formulated with legumes and vegetables.

Although the two types of meatballs did not differ significantly in total fat content, statistically significant differences were observed in saturated fat, the levels being significantly lower in the plant-based products (*p* < 0.001). As in plant-based sausages, the percentage of total fat that was saturated in the meatball analogues was on average less than 15%. The fats in the plant-based meatballs were mostly sunflower or rapeseed oil. Despite the indisputable nutritional advantage of vegetable oils, given their lower content of saturated fatty acids and trans fats compared to animal fats, their use in meat analogues has some disadvantages in terms of sensory properties. To achieve the characteristic marbled appearance of authentic meat products, which is due to the solid whitish animal fat, it may be necessary to use a vegetable oil richer in saturated fatty acids. In fact, coconut oil (rich in saturated fat) was the main lipid source in 10% of the plant-based meatballs surveyed. This fact is also important in the rest of plant-based categories. Another drawback of vegetable oils is an absence of specific volatile compounds often found in meat fat, which negatively effects the sensory attributes of the analogue product [29].

In contrast with the other food categories, the conventional meatballs had a statistically higher carbohydrate content than the plant-based substitutes (*p* < 0.001). The explanation is that, unlike burgers and sausages, meatballs are traditionally prepared by adding breadcrumbs to bind the dough. As is the case with burgers and sausages, plant-based meatballs had a higher mean total sugar content (2.2 g/100 g) than the animal-based equivalents (0.7 g/100 g), in which sugars represented a minimal percentage of the nutritional composition. In most of the plant-based meatballs surveyed sugar was not added as a separate ingredient but provided by vegetables such as carrots and beets. Cereals and legumes also contain a small proportion of simple sugars that contribute to the total content.

The total fiber content declared on the labels of the meatball analogues was highly variable (with fiber contents ranging from 1.5 to 14 g/100 g), corresponding to the wide range of vegetable ingredients used in their formulation. It should be noted that the ingredients contributing to the total fiber content in plant-based meatballs, as well as in the other plant-based food categories, were mainly products rich in soluble fiber with prebiotic effects, whose potential health benefits go beyond accelerating intestinal transit. These benefits may include the regulation of blood glucose or cholesterol levels and inducing satiation. However, this potential health outcome of consuming plant-based meat analogues has been little studied [30].

#### 3.1.4. Nuggets

When comparing the plant-based and conventional nuggets, no statistically significant differences were found in the average values of any nutritional parameter (Figure 5). However, the protein content in the different plant-based nuggets was extremely variable, with a high proportion of products containing insufficient protein to be considered as meat equivalents.

The plant-based nuggets had a higher average fat content than their meat counterparts, although the differences were not statistically significant (*p* > 0.05). To achieve a crunchy exterior, nuggets are coated in batter based on cereals and fat, which contributes greatly to the overall content of fat and carbohydrates, and therefore the energy value. This feature also reduces the differences in nutritional content between the plant- and animal-based nuggets; in fact, the differences were often greater between products of the same type. Notably, nuggets were the only food category without significant differences in sugar between plant- and animal-based products (1.2 g/100 g compared to 1.3 g/100 g, respectively).

Little information is available on the nutritional content of plant-based nuggets compared to those of animal origin. Bryngelsson et al. [15] observed that plant-based nuggets marketed in Sweden had lower total fat and protein than their meat counterparts but found no significant differences in energy value and content of saturated fat, salt, fiber, iron, folate, and vitamin B12.

### 3.2. Other Information on the Labels of Plant-Based Meat Analogues

One of the reasons for the growing consumption of these plant-based meat alternatives, besides ethical concerns for animal welfare and the environment, is the belief that they are healthier and less allergenic, being free of additives found in meat products, such as sulfites. However, our review of the ingredient lists revealed that most of the plant-based meat analogues contain one or more potential allergens, the most frequent being soy (in the form of beans, flour, protein concentrate or isolate, or tofu) and wheat (flour or gluten). Others are mustard, sulphites, nuts and, occasionally, milk and eggs.

Although many of these plant-based meat analogues contain wheat or gluten, each category included products that were apparently suitable for celiacs (40% of meatballs, 20% of burgers and sausages, and 16% of nuggets). The labels of 4% of sausages and 12% of nuggets clearly indicated they were gluten-free. Labels on many of the surveyed products indicated their suitability for vegetarians and/or vegans, although the logos or graphic images used for this purpose differed between brands.

A relatively high proportion of the plant-based products were specifically labelled as organic (80% of sausages, over 50% of burgers, 21% of meatballs, and 16% of nuggets). The increase in the consumption of organic products in recent years is associated with changing consumer lifestyles and health concerns, as well as a growing awareness of climate change and a desire to help protect the environment. However, the distant origin of some of the ingredients and the energy costs of food processing generate questions about the real environmental impact of plant-based meat analogues.

**Figure 6:**
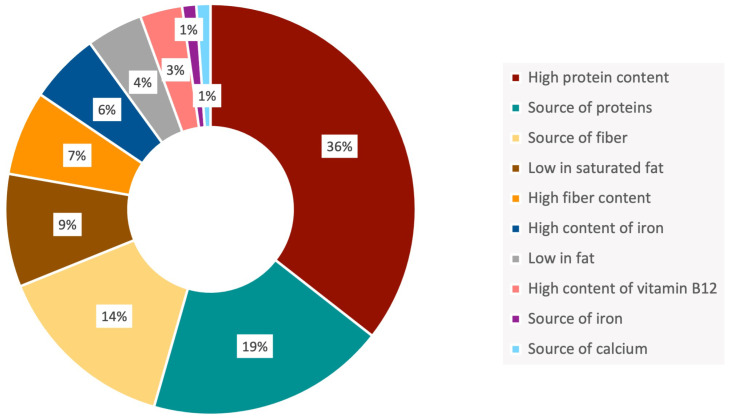
Nutritional claims on the labels of plant-based meat analogues.

None of the surveyed conventional meat products made nutritional or health claims on their labels, although some could probably incorporate them with only minor modifications in their formulation. On the contrary, a high percentage of the plant-based products made nutritional claims, burgers most frequently and nuggets the least. Among all the nutritional claims identified (Figure 6), those referring to the content of protein (“a source of protein” or “high protein content”) were the most common, followed by fiber (“a source of fiber” or “high in fiber”) and low fat or saturated fat. Despite the variability observed in ingredients, these three nutritional parameters are the ones that most differentiate the plant-based analogues from their meat counterparts.

A low proportion of plant-based meat analogues claimed to contain iron and vitamin B12, two micronutrients associated with a risk of deficiency in the vegetarian/vegan community. Although some meat substitutes were enriched in these two micronutrients, not all them declared it in the label. For example, soybeans contain leghemoglobin, which not only gives the food product a red color, but is also rich in heme iron, a component of meat that contributes to its distinctive flavor when cooked [30]. It should also be noted that none of the analyzed products declared zinc content on their labels.

Salome et al. [31] recently suggested that if the plant-based meat analogues available on the market contained sufficient iron and zinc, they could play a useful role in encouraging consumers to follow a healthier dietary pattern with less red meat intake. This would require the reformulation of products to improve their nutritional quality, choosing ingredients that are low in sodium and rich in beneficial nutrients.

Another important aspect to consider is the cost of plant- and animal-based products (Table 2). According to our evaluation, all four categories of plant-based meat analogues were more expensive than their meat equivalents, with mean prices (in euros per kg of product) being 1.6, 1.9, 2.2, and 2.9-fold higher for plant-based burgers, meatballs, nuggets and sausages, respectively. This fact can also have an impact in the acceptance of plant-based products and become a barrier for their widespread consumption.

In our work, only macronutrient content of the plant-based meat analogues was compared to their meat counterparts. It is important to bear in mind that animal-based foods are an important source of a range of essential micronutrients, such as iron and zinc, and the only source of vitamin B12. In this sense, a recent audit performed in Australian supermarkets highlighted that plant-based meat analogues contain less vitamin B12, iron, and zinc in comparison with their equivalent meat products [7]. In fact, unless fortified, plant-based meat analogues either do not contain or contain insignificant amounts of these nutrients. All in all, future studies should also include a thorough assessment of the micronutrients content of currently marketed plant-based meat analogues.

## 4. Conclusions

In summary, plant-based meat analogues vary greatly in energy and nutrient content, even within the same product category, with a coefficient of variation reaching or exceeding 100% in some cases. This variability is due to the wide range of ingredients and formulations used in their preparation. As a result, it is difficult to compare their nutritional content with that of conventional meat products or generalize about the level of nutritional equivalence.

Despite the considerable effort invested in designing and formulating meat analogues, not all the products contained significant amounts of meat-like protein. Soy was the most common source of protein, with other legumes, such as chickpeas or peas, used to a lesser extent. In many products, soy was supplemented with cereals, which can improve the quality of the protein, achieving a closer approximation to animal protein. On the other hand, the plant-based products generally contained less total and saturated fat and higher amounts of fiber and complex carbohydrates compared to the meat equivalents. They did not match the levels of essential nutrients such as iron, zinc, and vitamin B12 that are found in the meat products they seek to mimic. The salt content, while also highly variable, was generally lower in the plant-based products.

The development of plant-based meat alternatives that are both nutritious and tasty is a challenge for the food industry. Marketed as substitutes of existing meat products, they could be beneficial for public health and the environment as well as providing more choice for consumers. The nutritional profile of some plant-based meat analogues could be improved by modifying the formulation and ingredient selection, for example, ensuring a significant content of protein of high nutritional quality and of certain micronutrients, such as iron, zinc, and vitamin B12, and also by offering allergen-free options. Overall, the re-formulation of plant-based meat analogues should be made, taking into consideration healthy dietary patterns and issues of sustainability in sourcing ingredients.

## Figures and Tables

**Table 1:** Number of ingredients (mean, minimum, and maximum values) in plant- and animal-based burgers, sausages, meatballs, and nuggets.

	Burgers		Sausages		Meatballs		Nuggets	
	Plant-Based	Animal	Plant-Based	Animal	Plant-Based	Animal	Plant-Based	Animal
Mean	13.4	9.67	12.8	11.5	16	12.4	16	17.4
Range	9–22	6–14	7–15	10–14	8–24	10–16	10–29	14–23

**Table 2:** Price in euros per kg of product (mean, minimum, and maximum values) of plant- and animal-based burgers, sausages, meatballs, and nuggets.

	Burgers		Sausages		Meatballs		Nuggets	
	Plant-Based	Animal	Plant-Based	Animal	Plant-Based	Animal	Plant-Based	Animal
Mean	19.9	12.2	20.5	7.1	19.0	10.2	20.8	9.5
Range	9.9–31.3	6.5–23.2	9.3–31.6	2.4–21.1	13.1–24.0	5.4–15.9	10.0–31.9	4.4–19.8

## Supplementary Materials

The following supporting information can be downloaded at: https://www.mdpi.com/article/10.3390/nu15122807/s1, Table S1: Ingredients listed on the labeling of plant-based meat analogues in descending order of frequency.
